# Associations between air pollution and outpatient visits for arrhythmia in Hangzhou, China

**DOI:** 10.1186/s12889-020-09628-y

**Published:** 2020-10-08

**Authors:** Mingwei Wang, Juan Chen, Zhi Zhang, Ping Yu, Wentao Gan, Zhaoming Tan, Junzhe Bao

**Affiliations:** 1 Department of Cardiology, the Affiliated Hospital of Hangzhou Normal University, Hangzhou, China; 2 Nanjing Municipal Human Resources and Social Security Bureau, Nanjing, China; 3 College of Public Health, Zhengzhou University, Zhengzhou, China

**Keywords:** Arrhythmia, Air pollution, Time-series analysis, Season

## Abstract

**Background:**

Arrhythmia is a common cardiovascular event that is associated with increased cardiovascular health risks. Previous studies that have explored the association between air pollution and arrhythmia have obtained inconsistent results, and the association between the two in China is unclear.

**Methods:**

We collected daily data on air pollutants and meteorological factors from 1st January 2014 to 31st December 2016, along with daily outpatient visits for arrhythmia in Hangzhou, China. We used a quasi-Poisson regression along with a distributed lag nonlinear model to study the association between air pollution and arrhythmia morbidity.

**Results:**

The results of the single-pollutant model showed that each increase of 10 μg/m^3^ of Fine particulate matter (PM_2.5_), Coarse particulate matter (PM_10_), Sulphur dioxide (SO_2_), Nitrogen dioxide (NO_2_), and Ozone (O_3_) resulted in increases of 0.6% (− 0.9, 2.2%), 0.7% (− 0.4, 1.7%), 11.9% (4.5, 19.9%), 6.7% (3.6, 9.9%), and − 0.9% (− 2.9, 1.2%), respectively, in outpatient visits for arrhythmia; each increase of 1 mg/m^3^ increase of carbon monoxide (CO) resulted in increase of 11.3% (− 5.9, 31.6%) in arrhythmia. The short-term effects of air pollution on arrhythmia lasted 3 days, and the most harmful effects were observed on the same day that the pollution occurred. Results of the subgroup analyses showed that SO_2_ and NO_2_ affected both men and women, but differences between the sexes were not statistically significant. The effect of SO_2_ on the middle-aged population was statistically significant. The effect of NO_2_ was significant in both the young and middle-aged population, and no significant difference was found between them. Significant effects of air pollution on arrhythmia were only detected in the cold season. The results of the two-pollutants model and the single-pollutant model were similar.

**Conclusions:**

SO_2_ and NO_2_ may induce arrhythmia, and the harmful effects are primarily observed in the cold season. There is no evidence of PM_2.5_, PM_10_, CO and O_3_ increasing arrhythmia risk. Special attention should be given to sensitive populations during the high-risk period.

## Background

Cardio-cerebrovascular diseases and cancer are the main causes of death worldwide [1], and the associations between these diseases and environmental factors have been frequently reported [2, 3]. According to an analysis of the burdens of disease caused by air pollution conducted by the World Health Organization (WHO), over 2 million premature deaths each year can be attributed to urban outdoor and indoor air pollution [4]. In recent years, research on the association between air pollution and cardiovascular or respiratory diseases has received increased attention, and this work has generally shown that exposure to air pollutants is closely associated with the occurrence of cardiovascular and respiratory disease [5–9].

Arrhythmia is a common cardiovascular event and is associated with increased cardiovascular health risks [10], such as cardiac arrest (or death), resulting in reduced quality of life, disability, mortality, and increased medical care costs. One review has indicated that there is a high degree of uncertainty relating to the effect of air pollution on arrhythmias [11]. Most previous studies examining the relationship between air pollution and arrhythmias have been conducted in North America and Western Europe; therefore, the results of these studies may not apply to mainland China because of differences in the characteristics of air pollution, weather, health conditions, and the susceptibility of the population. In addition, evidence from developing countries is limited. The mechanism by which air pollutants cause acute cardiac events might involve cardiac autonomic function, myocardial repolarization, local and systemic inflammation, reactive oxygen species, coagulation, and myocardial ischemia [12].

In this study, we assumed that short-term exposure to air pollutants might induce arrhythmia and used a time-series quasi-Poisson model to study the association between the daily mean concentration of air pollutants and daily outpatient visits for arrhythmia. We aimed to identify the period of highest risk and characteristics of the sensitive population.

## Methods

### Study area

Hangzhou is the capital city of Zhejiang Province, an important political, economic, cultural, and financial center in eastern China (30°16′N, 120°12′E) and has a typical subtropical monsoon climate. The residential population of Hangzhou reached 9 million in 2015.

### Air pollution and meteorological data collection

Air pollution data were collected from the National City Air Quality Real-time Release Platform (http://106.37.208.233:20035/) and covered the period from 1st Jan. 2014 to 31st Dec. 2016, and the daily mean concentrations for each air pollutant were averaged for all of the 11 fixed-site air pollution monitoring stations. The meteorological data were collected from the National Meteorological Data Sharing Platform (http://data.cma.cn/) and consisted of daily mean temperature, maximum temperature, minimum temperature, mean relative humidity, mean wind speed, and atmospheric pressure.

### Outpatient arrhythmia data

Outpatient arrhythmia data were collected during the same period from the arrhythmia outpatient office at the Cardiac Centre of the Affiliated Hospital of Hangzhou Normal University (acquired from the HIS system of the hospital), including date of visit, age, gender, and home address. The diagnosis of arrhythmia was made by clinicians based on ECG standards. Appointments and follow-up patients were excluded. Patients from other areas (based on their home address) were also excluded, but these represented a small fraction of patients in the data set.

### Statistical analyses

A generalized linear model with a quasi-Poisson distribution was used to explore the association between air pollution and outpatient visits of patients with arrhythmia. The following covariates were included: calendar date, daily mean temperature, relative humidity, public holiday, and day-of-week. A natural cubic spline with seven degrees of freedom per year was applied to calendar time to smoothly model long-term and seasonal patterns [13]; another cubic spline with three degrees of freedom was applied to smoothly model daily mean ambient temperature (the 7-day moving average) and relative humidity [14]. Public holidays and the day-of-week were adjusted as indicator variables. We created a cross-basis matrix for air pollutants to consider their possible delayed and cumulative effects on arrhythmia, within the framework of the distributed lag-linear model [15]. The models were used to estimate the relative risks of outpatient visits for arrhythmia at various degrees of ambient air pollution: 10 μg/m^3^ for Fine particulate matter (PM_2.5_), Coarse particulate matter (PM_10_), Sulphur dioxide (SO_2_)_,_ Nitrogen dioxide (NO_2_), Ozone (O_3_); and 1 mg/m^3^ for carbon monoxide (CO).

Subgroup analyses were performed to identify populations sensitive to air pollution by calculating the relative risk of cardiac arrhythmia caused by air pollution in each subgroup. The warm season ran from May to October, and the cold season ran from November to April. We used the Cochran Q test to test for differences between subpopulations.

Sensitivity analyses were performed by changing df values for the time trend and relative humidity, as well as the lag days of air pollution in the model. All statistical analyses were computed using R software (version 3.4.0) with the “dlnm” package (version 2.3.2) to create distributed lag linear models. Two-tailed *p*-values < 0.05 were considered statistically significant.

## Results

A total of 16,191 cases of arrhythmia outpatients were included in this study, with female patients accounting for a slightly higher number of cases than males. Most patients were aged between 45 and 60 years old, and the number of visits in the hot season (May–October) was slightly greater than in the cold season (November–April) (Table 1).

**Table 1:** Demographic and seasonal characteristics of outpatient visits for arrhythmia

Variables	Number of Patients	Percentage (%)
Gender Groups		
Male	7206	44.5
Female	8985	55.5
Age Groups		
< 45	3701	22.9
45–60	7368	45.5
≥ 60	5122	31.6
Season Groups		
Warm season	8152	50.3
Cold Season	8039	49.7

The mean daily concentration (standard deviation, SD) of PM_2.5_, PM_10_, SO_2_, NO_2_, CO, and O_3_ were 52.9 (29.2) μg/m^3^, 77.6 (41.8) μg/m^3^, 15.5 (7.7) μg/m^3^, 43.9 (16.5) μg/m^3^, 0.85 (0.25) mg/m^3^ and 55.1 (27.1) μg/m^3^, respectively. The average daily readings (SD) of air temperature, relative humidity, air pressure, and wind speed were 17.8 (8.4) °C, 74.4 (13.9) %, 1011.4 (8.9) hPa, and 3.9 (3.8) m/s, respectively (Table 2).

**Table 2:** Distributions of daily air pollution, meteorological factors, and outpatient visits for arrhythmia

Variables	Mean + SD	Min	25th	50th	75th	Max
PM_2.5_ (ug/m^3^)	52.9 + 29.2	7.6	31.2	46.6	67.7	205.5
PM_10_ (ug/m^3^)	77.6 + 41.8	5.8	46.6	70.5	101.0	271.0
SO_2_ (ug/m^3^)	15.5 + 7.7	4.1	9.7	13.5	19.1	46.7
NO_2_ (ug/m^3^)	43.9 + 16.5	9.3	31.5	41.8	54.4	109.7
CO (mg/m^3^)	0.85 + 0.25	0.39	0.67	0.80	0.97	2.05
O_3_ (ug/m^3^)	55.1 + 27.1	5.3	34.2	51.8	73.5	149.3
Temperature (°C)	17.8 + 8.4	−5.0	10.5	19.1	24.4	34.4
Humidity (%)	74.4 + 13.9	27.0	65.0	76.0	85.0	98.0
Air pressure (hPa)	1011.4 + 8.9	989.3	1003.7	1011.1	1018.7	1036.6
Wind Speed (m/s)	3.9 + 3.8	0.0	0.5	3.2	7.4	12.7
Outpatient visits	14.8 + 7.9	0.0	9.0	14.0	20.0	59.0

We have examined the shape of the exposure-response relationships using natural spline functions and found them to be generally linear (Additional file 1). Therefore, generalized linear model with linear function was adopted. Table 3 showed that each increase of 10 μg/m^3^ in the concentration of atmospheric PM_2.5_, PM_10_, SO_2_, NO_2_, and O_3_ led to an increase in the number of arrhythmia outpatients (Lag of 0–3 d), with relative risks (RRs, 95% CI) of 1.006 (0.991, 1.022), 1.007 (0.996, 1.0017), 1.119 (1.045, 1.199), 1.067 (1.036, 1.099), and 0.991 (0.971, 1.012), respectively. The RR for a 1 mg/m^3^ increase in CO was 1.113 (0.941, 1.316). The effects of SO_2_ and NO_2_ were statistically significant, and the most harmful effect was observed on the same day that the pollution occurred. For each increment in the interquartile range of each pollutant, the RRs (95% CI) were 1.025 (0.970, 1.083), 1.038 (0.978, 1.100), 1.116 (1.041, 1.188), 1.160 (1.079, 1.303), 1.032 (0.982, 1.086), and 0.966 (0.889, 1.049) for PM_2.5_ (36.50 μg/m^3^), PM_10_ (54.39 μg/m^3^), SO_2_ (9.41 μg/m^3^), NO_2_ (22.93 μg/m^3^), CO (0.30 mg/m^3^), and O_3_ (39.26 μg/m^3^), respectively.

**Table 3:** Distributions of relative risks for arrhythmia caused by six air pollutants on lags of different lengths (in days)^a^

Lag	PM _2.5_	PM _10_	SO _2_	NO _2_	CO	O_3_
0	1.009 (0.998–1.020)	1.003 (0.995–1.011)	1.045 (0.997–1.095)	1.023 (0.999–1.047)	1.084 (0.943, 1.246)	0.997 (0.979, 1.015)
1	0.996 (0.984–1.008)	1.001 (0.993–1.010)	1.030 (0.986–1.076)	1.023 (0.999–1.048)	1.031 (0.890, 1.196)	0.999 (0.983, 1.015)
2	1.006 (0.994–1.018)	1.004 (0.996–1.013)	1.030 (0.987–1.075)	1.007 (0.999–1.030)	1.023 (0.886, 1.826)	0.996 (0.981, 1.012)
3	0.994 (0.984–1.005)	0.997 (0.989–1.004)	1.009 (0.969–1.051)	1.012 (0.992–1.033)	0.919 (0.804, 1.049)	0.993 (0.980, 1.007)
0–3	1.006 (0.991–1.022)	1.007 (0.996–1.017)	1.119 (1.045–1.199)	1.067 (1.036–1.099)	1.113 (0.941, 1.316)	0.991 (0.971, 1.012)

^a^ For PM_2.5_, PM_10_, SO_2_, NO_2_, and O_3_, the relative risks were associated with increments of 10 μg/m^3^; for CO, the increment was 1 mg/m^3^

The subgroup analyses showed that SO_2_ and NO_2_ affected both male and female patients, and there was no significant difference in their effects. The effect of SO_2_ was statistically significant in the middle-aged group, and the effect of NO_2_ was statistically significant in both the younger age and the middle-aged groups, while the difference between them was not statistically significant. The effect of air pollution on arrhythmia was only statistically significant in the cold season (Table 4). The results of the two-pollutant model and the single-pollutant models were similar (Table 5).

**Table 4:** Distributions of the relative risks for arrhythmia at various levels of air pollutants in different subgroups and seasons ^a^

Variables	PM_2.5_	PM_10_	SO_2_	NO_2_	CO	O_3_
Overall	1.007 (0.992, 1.022)	1.007 (0.997, 1.018)	1.119 (1.045, 1.199)	1.067 (1.037, 1.099)	1.113 (0.941, 1.316)	0.991 (0.971, 1.012)
Gender						
Male	1.018 (0.999, 1.037)	1.009 (0.996, 1.023)	1.112 (1.028, 1.201)	1.071 (1.032, 1.112)	1.251 (1.013, 1.546)	0.989 (0.963, 1.016)
Female	0.997 (0.979, 1.016)	1.006 (0.993, 1.018)	1.137 (1.032, 1.210)	1.064 (1.027, 1.102)	1.011 (0.824, 1.239)	0.993 (0.968, 1.018)
Age						
< 45	1.018 (0.990, 1.048)	1.013 (0.993, 1.033)	1.105 (0.973, 1.255)	1.109 (1.050, 1.171)	1.052 (0.763, 1.450)	0.989 (0.954, 1.027)
45–60	1.007 (0.988, 1.026)	1.010 (0.997, 1.023)	1.154 (1.062, 1.255)	1.076 (1.038, 1.116)	1.184 (0.962, 1.457)	0.984 (0.958, 1.010)
≥ 60	0.995 (0.972, 1.019)	0.998 (0.982, 1.014)	1.077 (0.963, 1.204)	1.027 (0.981, 1.076)	1.013 (0.781, 1.313)	1.004 (0.970, 1.038)
Season						
Warm	0.989 (0.963, 1.015)	0.994 (0.977, 1.011)	1.052 (0.924, 1.195)	0.995 (0.944, 1.048)	0.944 (0.698, 1.276)	0.997 (0..962, 1.033)
Cold	1.016 (0.997, 1.033)	1.013 (0.999, 1.026)	1.122 (1.034, 1.223)	1.075 (1.033, 1.119)	1.142 (0.903, 1.443)	0.982 (0.925, 1.042)

^a^For PM_2.5_, PM_10_, SO_2_, NO_2_, and O_3_, the relative risks were associated with increments of 10 μg/m^3^; for CO the increment was 1 mg /m^3^. A Cochran Q test was used to test for differences between subpopulations, and no statistically significant differences were found between subpopulations

**Table 5:** Results of the two-pollutant model

Controlled Pollutant	PM_2.5_	PM_10_	SO_2_	NO_2_	CO	O_3_
None	1.007 (0.992,1.022)	1.007 (0.997,1.018)	1.119 (1.045,1.199)	1.067 (1.037,1.099)	1.113 (0.941,1.316)	0.991 (0.971,1.012)
PM_2.5_	/	1.002 (0.984,1.019)	1.125 (1.051,1.203)	1.079 (1.042,1.116)	1.076 (0.862,1.345)	0.990 (0.969,1.011)
PM_10_	1.008 (0.984,1.032)	/	1.131 (1.056,1.219)	1.086 (1.048,1.124)	1.093 (0.984,1.338)	0.991 (0.970,1.012)
SO_2_	0.999 (0.984,1.016)	1.001 (0.989,1.013)	/	1.061 (1.027,1.096)	1.068 (0.880, 1.317)	0.990 (0.969,1.011)
NO_2_	0.996 (0.979,1.012)	0.997 (0.985,1.009)	1.084 (1.009,1.164)	/	1.031 (0.825, 1.297)	0.992 (0.971,1.013)
CO	1.003 (0.985,1.022)	1.004 (0.992,1.016)	1.125 (1.057,1.198)	1.066 (1.034,1.098)	/	0.992 (0.971,1.013)
O_3_	1.009 (0.994,1.025)	1.008 (0.998,1.019)	1.130 (1.061,1.200)	1.061 (1.031,1.089)	1.120 (0.946,1.323)	/

The sensitivity analyses showed that the relative risk for cardiac arrhythmia was generally similar for various degrees of freedom for the time trend (5–9 df/year), humidity (2–5 df), as well as for various lag times (2–14 days) (Additional file 2, Additional file 3). This finding confirmed the robustness of our results.

## Discussion

We found that SO_2_ and NO_2_ had significant effects on arrhythmia outpatient visits in Hangzhou. A previous meta-analysis has revealed significant associations between arrhythmia hospitalization or arrhythmia mortality and PM_2.5_, PM_10_, SO_2_, and NO_2_, after synthesizing the results of 23 studies. Furthermore, these associations were stronger in Asia compared with those that have been documented for Europe and North America. Most of the studies included in this meta-analysis were conducted in developed countries, with only one conducted in China, and this study had not considered the lag effect [16]. Link et al. also found a positive association between short-term PM_2.5_ exposure and atrial fibrillation [17] by utilizing a case-crossover analysis of 176 patients. However, a time-series study of nearly 400,000 emergency department visitors from seven Canadian cities found that there was no significant association between air pollution and arrhythmia [18]. Bunch et al. also found no significant association between atrial fibrillation hospitalization and PM_2.5_ [19]. We found that increased concentrations of PM_2.5_ and PM_10_ might increase the risk of arrhythmia, but the effects were not statistically significant. This lack of significance may stem from the small sample size and the limited number of years that were sampled in this study.

Our study revealed that air pollution affects both sexes and primarily affects the middle-aged population; however, there were no statistically significant differences between sexes and age groups. Zhao et al. also found that air pollution affected both males and females, with a more pronounced deleterious effect on females [20]. A study in Shanghai found that elderly patients with arrhythmia were more sensitive to air pollution [20], while a meta-analysis involving 25 studies found that the health effects of air pollution on elderly patients were not any greater than their effects on the general population [16]. The physical condition of the middle-aged population is generally better than that of the elderly. However, middle-aged patients are often actively working and experience more stress; in contrast, elderly patients are often retired and are thus not working under as stressful conditions. Consequently, when the air pollution is severe, elderly patients have the option of simply not leaving their homes. These factors may explain why middle-aged patients are susceptible to air pollution. Because of the small sample size and the limited number of years that were examined, the lack of a significant association between air pollution and arrhythmia in elderly patients requires additional research.

Our study found that the harmful effect of air pollution on arrhythmia primarily occurred in the cold season. Santurtún et al. also found that the effect of NO_2_ on arrhythmia primarily occurred in the winter [21]. In contrast, Zhao et al. found a stronger association between NO_2_, SO_2_, and cardiac arrhythmias in the warm season [20]. The reasons underlying the greater harmful effects of air pollution in the cold season may be that the concentration of air pollutants, and thus the potential for damage, is higher in the cold season; that the combined effects of low temperature and air pollution.

One of the possible pathways connecting short-term exposure to air pollution and cardiovascular events could be autonomic nervous system dysfunction [22]; however, toxicological studies in animals and clinical studies in humans are needed to verify this hypothesis. Air pollutants have also been found to be associated with increased systemic inflammation, increased platelet activation, and decreased erythrocyte antioxidant enzyme activity; all of these factors may induce arrhythmias [21].

We found that there were no statistically significant differences between the effects of air pollution in different subgroups. This may be due to the fact that the difference between different subgroups is only caused by sampling error; or it may be that the number of samples involved in this study is not enough, and the significance of the difference has not been detected. These contents need further study in the future.

There are several limitations of this study requiring consideration. First, given that this was an ecological study, ecological inaccuracies may exist. The air pollution and meteorological data were obtained from several monitoring stations instead of individual exposure measurements; therefore, the exposure assessment in this study might be inaccurate. Second, given the limitations of data acquisition, socioeconomic factors were not considered in this study; in addition, outpatients could not be classified into disease subtypes, which could only be classified for inpatients, according to the World Health Organization’s International Classification of Diseases, the 10th version (ICD-10). Third, this study was conducted in a single city (Hangzhou); thus, our findings cannot be generalized to other cities with different environmental and economic characteristics. Cohort studies are needed to assess the long-term effects of air pollution on arrhythmias.

## Conclusions

SO_2_ and NO_2_ may induce arrhythmia, and the harmful effects are primarily observed in the cold season. Air pollution affects both males and females, and the middle-aged population is especially sensitive to air pollution. The government should take action to reduce air pollution and make special interventions for sensitive individuals during the cold season.

## Supplementary information

**Additional file 1:** The exposure-response relationships between air pollutants and arrhythmia using natural spline functions.

**Additional file 2:** The change of relative risk for arrhythmia caused by NO_2_ in various degrees of freedom.

**Additional file 3:** The change of relative risk for arrhythmia caused by NO_2_ in various lag time.

## Data Availability

Due to confidentiality requirements, the data involved in this study is currently not publicly available.

## Abbreviations

PM_2.5_: Fine particulate matter
PM_10_: Coarse particulate matter
SO_2_: Sulphur dioxide
NO_2_: Nitrogen dioxide
CO: Carbon monoxide
O_3_: Ozone
WHO: World Health Organization
ICD-10: International Classification of Diseases, the 10th version
SD: Standard deviation, SD
RR: Relative risk
CI: Confidence interval
