# Supplementary material for: Associations between air pollution and outpatient visits for arrhythmia in Hangzhou, China
Source: BMC Public Health. 2020 Oct 8;20:1524. doi: 10.1186/s12889-020-09628-y (PMC7542945; doi:10.1186/s12889-020-09628-y)
Supplement: Supplementary file 1 — Additional file 1: The exposure-response relationships between air pollutants and arrhythmia using natural spline functions. [file 12889_2020_9628_MOESM1_ESM.docx]

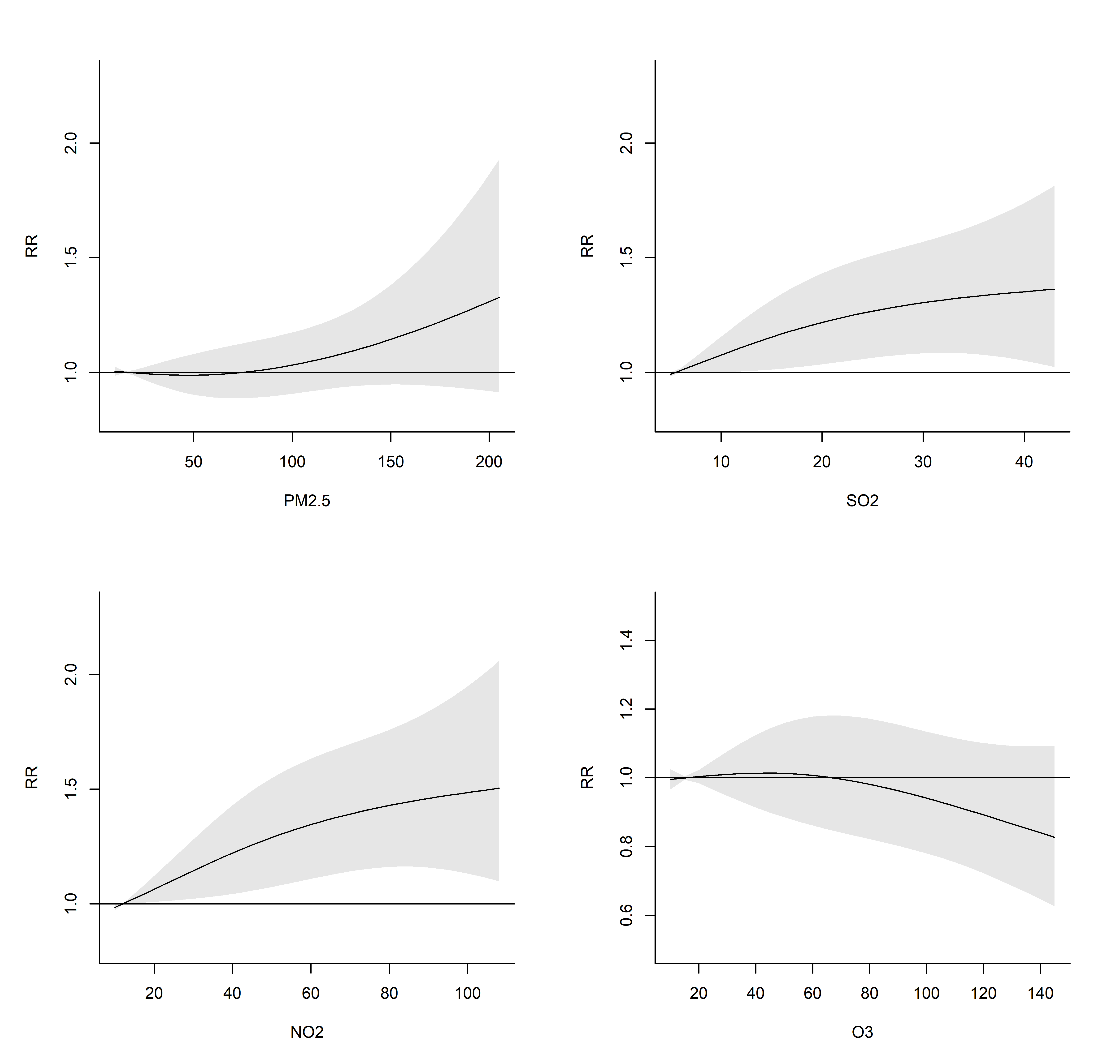


The exposure-response relationships between air pollutants and arrhythmia using natural spline functions
