# Supplementary material for: Associations between air pollution and outpatient visits for arrhythmia in Hangzhou, China
Source: BMC Public Health. 2020 Oct 8;20:1524. doi: 10.1186/s12889-020-09628-y (PMC7542945; doi:10.1186/s12889-020-09628-y)
Supplement: Supplementary file 2 — Additional file 2: The change of relative risk for arrhythmia caused by NO2 in various degrees of freedom. [file 12889_2020_9628_MOESM2_ESM.docx]

| The change of relative risk for arrhythmia caused by NO_2_ in various degrees of freedom | | | |
| --- | --- | --- | --- |
| df (time/year) | df (humidity) | RR | 95% eCI |
| 5 | 2 | 1.066 | 1.037 – 1.096 |
|  | 3 | 1.065 | 1.036 – 1.095 |
|  | 4 | 1.066 | 1.036 – 1.096 |
|  | 5 | 1.066 | 1.037 – 1.096 |
| 6 | 2 | 1.071 | 1.040 – 1.102 |
|  | 3 | 1.071 | 1.040 – 1.102 |
|  | 4 | 1.071 | 1.040 – 1.102 |
|  | 5 | 1.071 | 1.041 – 1.103 |
| 7 | 2 | 1.067 | 1.037 – 1.099 |
|  | 3 | 1.067 | 1.037 – 1.099 |
|  | 4 | 1.067 | 1.037 – 1.099 |
|  | 5 | 1.068 | 1.037 – 1.100 |
| 8 | 2 | 1.062 | 1.031 – 1.095 |
|  | 3 | 1.063 | 1.031 – 1.095 |
|  | 4 | 1.063 | 1.031 – 1.095 |
|  | 5 | 1.063 | 1.032 – 1.096 |
| 9 | 2 | 1.062 | 1.029 – 1.095 |
|  | 3 | 1.062 | 1.029 – 1.095 |
|  | 4 | 1.062 | 1.029 – 1.096 |
|  | 5 | 1.063 | 1.030 – 1.097 |
|  |  |  |  |
