# Supplementary material for: Associations between air pollution and outpatient visits for arrhythmia in Hangzhou, China
Source: BMC Public Health. 2020 Oct 8;20:1524. doi: 10.1186/s12889-020-09628-y (PMC7542945; doi:10.1186/s12889-020-09628-y)
Supplement: Supplementary file 3 — Additional file 3: The change of relative risk for arrhythmia caused by NO2 in various lag time. [file 12889_2020_9628_MOESM3_ESM.docx]

| The change of relative risk for arrhythmia caused by NO_2_ in various lag time | | |
| --- | --- | --- |
| Lag time (days) | RR | 95% CI |
| 0-2 | 1.059 | 1.032 – 1.086 |
| 0-3 | 1.067 | 1.037 – 1.099 |
| 0-5 | 1.074 | 1.039 – 1.110 |
| 0-7 | 1.066 | 1.027 – 1.105 |
| 0-9 | 1.075 | 1.033 – 1.118 |
| 0-14 | 1.065 | 1.015 – 1.117 |
|  |  |  |
